# Antibiotic additive and synergistic action of rutin, morin and quercetin against methicillin resistant *Staphylococcus aureus*

**DOI:** 10.1186/s12906-015-0580-0

**Published:** 2015-03-12

**Authors:** Muhammad Usman Amin, Muhammad Khurram, Baharullah Khattak, Jafar Khan

**Affiliations:** Department of Microbiology, Kohat University of Science and Technology, Kohat, KP Pakistan; Department of Pharmacy, Shaheed Benazir Bhutto University, Sheringal, Dir Upper, KP Pakistan

**Keywords:** Morin, Rutin, Quercetin, MRSA

## Abstract

**Background:**

To determine the effect of flavonoids in conjunction with antibiotics in methicillin resistant *Staphylococcus aureus* (MRSA) a study was designed. The flavonoids included Rutin, Morin, Qurecetin while antibiotics included ampicillin, amoxicillin, cefixime, ceftriaxone, vancomycin, methicillin, cephradine, erythromycin, imipenem, sulphamethoxazole/trimethoprim, ciprofloxacin and levolfloxacin. Test antibiotics were mostly found resistant with only Imipenem and Erythromycin found to be sensitive against 100 MRSA clinical isolates and *S. aureus* (ATCC 43300). The flavonoids were tested alone and also in different combinations with selected antibiotics.

**Methods:**

Antibiotics and flavonoids sensitivity assays were carried using disk diffusion method. The combinations found to be effective were sifted through MIC assays by broth macro dilution method. Exact MICs were determined using an incremental increase approach. Fractional inhibitory concentration indices (FICI) were determined to evaluate relationship between antibiotics and flavonoids is synergistic or additive. Potassium release was measured to determine the effect of antibiotic-flavonoids combinations on the cytoplasmic membrane of test bacteria.

**Results:**

Antibiotic and flavonoids screening assays indicated activity of flavanoids against test bacteria. The inhibitory zones increased when test flavonoids were combined with antibiotics facing resistance. MICs of test antibiotics and flavonoids reduced when they were combined. Quercetin was the most effective flavonoid (MIC 260 μg/ml) while morin + rutin + quercetin combination proved most efficient with MIC of 280 + 280 + 140 μg/ml. Quercetin + morin + rutin with amoxicillin, ampicillin, cephradine, ceftriaxone, imipenem, and methicillin showed synergism, while additive relationship was indicated between morin + rutin and amoxicillin, cephradine, ceftriaxone, imipenem, and methicillin. Quercetin alone had an additive effect with ampicillin, cephradine, ceftriaxone, imipenem, and methicillin. Potassium leakage was highest for morin + rutin + quercetin that improved further in combination with imipenem. Morin and rutin alone had no activity but in combination showed activity against test bacteria.

**Conclusions:**

The flavonoids when used in combination with antibiotics were found to increase each other activity against test bacteria. The relationship between the flavonoids and antibiotics in most of the cases was additive. However in a few cases synergism was also observed. Flavonoids alone or in combinations also damaged bacterial cell membrane.

## Background

Plant secondary metabolites are characteristic of certain plant species that occur as part of their normal metabolism with little or no function in plant life cycle. These metabolites may have bioactivities and also medicinal value like anti-infective, antitumor, antithrombotic, and antihyperlipidemic [1]. Different species of genus Cipadessa, are used in mitigation of rheumatism, malaria, dysentery, diabetes, and haemorrhoids and as anti-venom in snake bites, with active constituents including flavonoids, terpenoids, tetranortriterpenoids, steroids and sesqueterpenoids [2]. This is indicative that these metabolites may be a common feature among different species of a genus, showing common pharmacological effects in different ailments. About 400 species of *Saussurea* genus, are used as traditional medicines in China and Tibet in problems related to menstruation, blood circulation, fever and rheumatoid arthritis. The reason for their use in these conditions is due to the presence of biologically active compounds including flavonoids, terpenoids, phytosterols, and phenolics. The antimicrobial activities in these plant species have been related to flavanoids, terpenoids and tannins [3]. Flavonoids are generally present in photosynthesizing cells [4]. From ancient times, preparations containing these compounds have been used to treat human diseases. This class of natural products is becoming the main target of research due to their antimicrobial potentials.

Flavonoids have good antibacterial activities, like chrysin that showed bacteriostatic activity against different Gram-negative bacteria such as *Escherichia coli* and *Pseudomonas aeruginosa*. Similarly, biacalien possess inhibitory effect on *S. aureus* and on the growth of spore forming *Bacillus subtilis*. Other flavonoids such as luteolin, lucenin, apigenin, saponarin and vitexin were effective against Gram-negative bacteria *Enterobacter cloacae*, *Klebsiella pneumonia*, *Proteus mirabilis*, *E. coli, P. aeruginosa,* and *Proteus vulgaris* [5]. An 126 interesting feature of these flavonoids is their potentiating and synergistic behaviour that is observed between active flavonoids as well as combination of flavonoids with antimicrobial agents [6]. Flavonoids like myricetin, datiscetin, kaempferol, qurecetin and flavones like luteolin have inhibited growth of MRSA and with myricetin were found effective against VRE as well [7]. In another study alkyl gallates (methyl, ethyl, propyl and butyl gallates) and gallic acid with different classes of antibiotics such as β-lactams (penicillin G, ampicillin, oxacillin, cephradine), quinolone (norfloxacin), aminoglycosides (streptomycin, kanamycin, vancomycin), chloramphenicol, arbekacin, fosfomycin and tetracycline were used in combinations against drug sensitive and resistant bacteria. It was observed that these combinations had maximum inhibitory activity in 90% clinical isolates at MIC of 15.6 μg/ml. However, combinations of β-lactams and alkyl gallates showed synergestic activities against MRSA and MSSA [8].

Resistance to antimicrobial agents is a global issue with methicillin resistant *Staphylococcus aureus* (MRSA) a major concern [9]. In a study conducted in Pakistan during 2005–2007, about 501 MRSA clinical isolates were isolated from skin and soft tissue infections and were tested for their susceptibilities against conventional antibiotics such as clindamycin, tetracyclines, cotrimoxazole and rifampicin, chloramphenicol and fusidic acid. All of these drugs were ineffective against MRSA isolates [10].

Difficulties in treatment of resistant microbes serve a challenge to discover new drugs that can be effective against these resistant bugs. Since plant metabolites are not part of conventional therapy they can be considered as monotherapy or in combination therapy against them*.* Keeping in view the emerging threat of MRSA, present study was designed to evaluate impact of selected flavonoids (morin, rutin, quercetin) alone and in combination with conventionally used antibiotics for their activities against *S. aureus* (ATCC 43300) and 100 MRSA clinical isolates.

## Methods

### Materials

The antibiotic discs included amoxicillin (AMO; 25 μg), ampicillin (AMP; 10 μg), ceftriaxone (CET; 30 μg), cefixime (CEF; 5 μg), cephradine (CEPH; 30 μg), erythromycin (ERY; 15 μg), vancomycin (VAN; 30 μg), methicillin (ME; 10 μg), ciprofloxacin (CIP; 5 μg), levolfloxacin (LEV; 5 μg), sulfamethaxozole-trimethoprim (S-T; 25 μg) and imipenem (IMP; 10 μg) were from Oxoid, UK while blank discs were purchased from Himedia, India. Test flavonoids; rutin, morin, and quercetin were purchased from Sigma-Aldrich, UK. Stock solutions of flavonoids morin, rutin, and quercetin were made at concentrations of 50 g/l, 6 g/l, and 10 g/l respectively, using ethanol. Bacterial culturing medias such as nutrient agar (NA; CM0003B), muller hinton agar (MHA; CM0337B), nutrient broth (N.B; CM0001B) and muller hinton broth (MHB; CM0405B) were from Oxoid, UK. mannitol salt agar (MSA; LAB007) was obtained from Lab M Limited, UK.

### Bacterial cultures collection, transport and processing

Clinical isolates (n = 300) were obtained from the microbiology laboratories of tertiary care hospitals which are Hayatabad Medical complex, Lady Reading Hospital and Khyber Teaching Hospital of Peshawar, KPK, Pakistan while *S. aureus* (ATCC 43300, Rockville, USA) present at PCSIR laboratories, was used as standard. Clinical isolates were transported to Microbiology lab, PCSIR Peshawar, Pakistan, for culturing, the same day within 2 hours after collection.

Clinical isolates were sub-cultured on sterile nutrient agar (NA) plates and then incubated at 37 ± 1°C for 18 – 20 hours. Following incubation, plates showing growth were subjected to Gram staining, catalase test, coagulase test [11], and mannitol salt agar differentiation [12]. The organisms showing yellow colonies on MSA plates, gram-positive cocci in clusters, coagulase + ve, and catalase + ve were considered *S. aureus*.

Isolated colonies of *S. aureus* from MSA plates were aseptically inoculated in sterile nutrient broth and incubated overnight at 37°C. Thereafter, turbidity of inoculum was adjusted to 0.5 McFarland using 0.9% (w/v) sterile normal saline and was used to prepare bacterial lawns on sterile MHA plates. Methicillin discs were applied on seeded plates and incubated overnight at 37 ± 1°C. Following incubation, plates with zones of inhibitions <10 mm diameter or no zone of inhibition were considered to be MRSA. The study was approved by the Ethical Review Committee of Kohat University of Science and Technology with a waiver of informed consent.

### Antibiotic sensitivity assays

Susceptibilities of clinical isolates and ATCC 43300 against selected antibiotics, flavonoids and antibiotic + flavonoids combinations were found using disc diffusion assay [13]. Flavonoids were tested alone, in different combinations, and with test antibiotics at different concentrations as shown in Table 1. Susceptibilities assays were carried using sterile MHA plates. Following overnight incubation at 37°C zones of inhibitions were recorded. Test substances were considered to have activity if zone of inhibition was > 10 mm, while ≤ 10 mm zone of inhibition were regarded as inactive.

**Table 1:** **Flavonoids used in antibiotic sensitivity assays**

**Flavonoids**	**Test concentrations used**
Morin (M)	100 μg, 200 μg, 300 μg, 400 μg, 500 μg
Rutin (R)	100 μg, 200 μg, 300 μg, 400 μg, 500 μg
Quercetin (Q)	100 μg, 200 μg, 300 μg

### Minimum inhibitory concentration (MIC) assays

MIC assays were carried out using broth macro-dilution method. Different concentrations of flavonoids, their combinations alone and with antibiotics were used. Flavonoids and their combinations included morin + rutin (M + R), quercetin (Q) and morin + rutin + quercetin (M + R + Q). Test antibiotics with flavonoids alone and their combinations included amoxicillin, ampicillin, ceftriaxone, cephradine, imipenem and methicillin. Test concentrations of flavonoids, flavonoid combinations alone and with antibiotics are given in Table 2. The MICs of these antibiotics against MRSA alone and in combination with flavonoids were determined to confirm the synergistic or additive effects. For the MIC’s assays stock solutions of these antibiotics were prepared using sterile distilled water.

**Table 2:** **Test concentrations of flavonoids and their combination for MIC assays**

**Flavonoids**	**Concentration ranges used for MIC assays (in μg/ml)**			
	**Broth half dilution method**		**Incremental increase approach †**	
	***Maximum***	***Minimum***	***Maximum***	***Minimum***
Morin (M)	NT*	NT	NT	NT
Rutin(R)	NT	NT	NT	NT
Quercetin(Q)	600	75	300	180
Morin + Rutin(M + R)	800 + 800	100 + 100	500 + 500	380 + 380
Morin + Rutin + Quercetin (M + R + Q)	600 + 600 + 400	150 + 150 + 100	400 + 400 + 260	260 + 260 + 120

† To determine Exact MIC’s of test substances an incremental increase approach was adopted with 20 μg decrease in each dilution.

*Not tested.

Exact MIC’s of flavonoids alone, in combinations and with test antibiotics were determined by an incremental increase approach in which higher concentrations were obtained by the addition of 20 μg increments to the lower concentrations. After test materials were transferred into sterile test tubes, bacterial cultures were prepared as mentioned earlier. After inoculation and addition of test materials tubes were incubated overnight at 37°C. Absence of any visible growth in test tubes was considered as the MIC of test material.

### Fractional Inhibitory concentration (FIC) & FIC Index

To determine the effectiveness of test substances for synergistic, antagonistic or additive effects FIC indices were measured, using following formulas [14].

- FIC of antibiotic (FIC_antibiotic_) = MIC of antibiotic in combination / MIC of antibiotic alone
- FIC of flavonoid (FIC_flavonoid_) = MIC of flavonoid in combination / MIC of flavonoid alone
- While FIC index was the sum of FIC of both antibiotic and flavonoid.
- FIC Index (FICI) = FIC_antibiotic_ + FIC_flavonoid_

In case of two or more flavonoids used in combination FIC was calculated in accordance to following formula [15].

$$ \mathrm{F}\mathrm{I}\mathrm{C}=\left(\mathrm{A}/\mathrm{Ma}\right)+\left(\mathrm{B}/\mathrm{Mb}\right) $$

Where A and B are the MIC’s of these compounds in combination while Ma and Mb are individual MICs of test compounds. The test materials in their combinations alone or with standard antibiotics were graded to have synergistic (≤0.5), additive (>0.5 to 1), indifferent (>1 to <2) or antagonistic (≥2) on the basis of FIC indices [16].

### Detection of cytoplasmic membrane damage

The assessment of the potassium present in medium was carried out using flame photometer (PFP7, Jenway, Sweden) at wavelength of 766.480 nm. Instrument was calibrated using standard solutions containing 0.05, 0.1, 0.5, 1.0 and 5.0 μg/ml potassium chloride in ultra-pure deionised water obtained from HACH water system USA.

Aliquots of 100 μl from each MRSA clinical isolate and *S. aureus* (ATCC 43330) were separately incubated overnight after incorporation of 1 ml previously sterilized nutrient broth. The rise in the amount of potassium in supernatant, caused by antibiotics, flavonoids, flavonoids-antibiotics combination, in clinical isolates and control strain was measured following separation of cellular debris by centrifugation at 4000 rpm.

## Results

### Antibiotic sensitivity assays

Quercetin, M + R, and M + Q + R showed some activities against MRSA clinical isolates and ATCC 43300. However, morin, rutin and Q + R, Q + M combinations were found inactive against test bacteria (Table 3). Quercetin and active combinations were found to be more effective when the antibiotics were combined with them. Antibiotics like AMO, AMP, CEPH, CET, ME, S-T, and CEF that were inactive when tested alone, expressed activity when combined with Q, M + R and M + R + Q (Table 4). However, test flavonoids were found to be having no impact on VAN and ERY activity, while causing reduction in CIP and LEV activities.

**Table 3:** **Average Zone of Inhibitions (in millimeters ± STDEV) of Morin, Rutin, Quercetin, Morin + Rutin, Quercetin + Rutin, Quercetin + Morin, and Morin + Quercetin + Rutin against MRSA clinical isolates and** ***S. aureus*** **(ATCC 43300)**

**Test flavonoid or flavonoids combination**	***S. aureus***	**MRSA clinical isolates (n = 100)**
M	0	0
R	0	0
Q	13.5 ± 0.21	13.33 ± 0.26
M + R	11.5 ± 0.22	11.58 ± 0.21
Q + R	0	0
Q + M	0	0
M + Q + R	16.5 ± 0.21	16.23 ± 0.26

**Table 4:** **Average zone of inhibitions (in mm ± STDEV) of antibiotics alone and with flavonoid/(s) against** ***S. aureus*** **(ATCC 43300) and MRSA clinical isolates (n =100)**

	**Antibiotic alone**	**M + R with antibiotic**	**Q with antibiotic**	**M + R + Q with antibiotic**
**AMO †**	0	14.5 ± 0.29	13.5 ± 0.21	23.50 ± 1.1
AMO *	0	14.18 ± 0.36	13.33 ± 0.26	23.73 ± 1.1
**AMP †**	0	11.5 ± 0.22	17 ± 0.29	22.5 ± 1.2
AMP *	0	11.58 ± 0.21	17.33 ± 0.30	22.63 ± 1.2
**CEPH†**	0	14.5 ± 0.32	18.5 ± 0.31	24.55 ± 1.00
CEPH*	0	14.18 ± 0.29	18.33 ± 0.30	24.22 ± 1.00
**CET †**	0	16.5 ± 0.30	20.5 ± 1.00	27 ± 1.20
CET*	0	16.18 ± 0..29	20.83 ± 1.00	27.24 ± 1.23
**IMP †**	16 ± 0.90	19.5 ± 0.58	22 ± 1.10	28 ± 1.00
IMP*	16.18 ± 0.92	19.62 ± 0.31	22.18 ± 1.12	28.21 ± 0.90
**ME †**	0	13.5 ± 0.31	16.5 ± 0.28	21.5 ± 1.1
ME*	0	13.18 ± 39	16.89 ± 0.29	21.73 ± 1.0
**VAN †**	18 ± 0.59	18 ± 0.59	18 ± 0.59	18 ± 0.59
VAN*	17.37 ± 0.80	17.37 ± 0.80	17.37 ± 0.80	17.37 ± 0.80
**LEV †**	14 ± 0.91	9 ± 0.25	6.5 ± 0.21	3 ± 0.24
LEV*	13.88 ± 1.21	9.52 ± 0.25	6.85 ± 0.22	3.23 ± 0..25
**CIP†**	10.5 ± 2.5	7.24 ± 0.19	4 ± 0.22	2.25 ± 0.24
CIP*	13.1 ± 3.1	8.1 ± 0.18	4.35 ± 0.24	2.73 ± 0.21
**ERY †**	22 ± 1.36	22 ± 1.36	22 ± 1.36	22 ± 1.36
ERY*	20.96 ± 2.1	20.96 ± 1.10	20.96 ± 1.10	20.96 ± 1.1
**S-T †**	0	11.5 ± 0.22	13 ± 0.21	14 ± 0.21
S-T*	0	11.58 ± 0.21	13.33 ± 0.26	14.23 ± 0.26
**CEF †**	0	11.5 ± 0.22	13 ± 0.21	14 ± 0.21
CEF*	0	11.58 ± 0.21	13.33 ± 0.26	14.23 ± 0.26

† activity against S. aureus (ATCC 43300).

*activity against clinical isolates.

The concentration at which M + R showed activity against the bacteria under study was 500 μg for each of the flavonoid. The inhibition zones of this combination observed at this concentration were 11.5 ± 0.22 mm against standard bacteria and 11.58 ± 0.21 mm against 100 clinical isolates. Moreover, M + R was found to increase activity of AMO, CEPH, IMP, CET and ME. CET activity was increased highest, from 0 to 16.5 ± 0.30 mm against standard and the zone of inhibition was increased from 0 to 16.5 ± 0.29 mm, in comparison to all other test antibiotics finding resistance against both standard strain and clinical isolates. The zone of inhibition of vancomycin and erythromycin were 18 ± 0.59 mm and 22 ± 1.36 mm against ATCC 43300, which remained same in combination with M + R. However, IMP activity enhanced from 16 ± 0.90 mm to 19.5 ± 0.58 mm against ATCC 43300, while for clinical isolates it became 19.62 ± 0.31 mm from 16.18 ± 0.92 mm in combination with M + R. The S-T and AMP had no activity against test bacteria alone as well as in combination with flavonoids. Ethanol, which was used in making solutions of flavonoids, was having no inhibitory activity.

Quercetin was found to be more active than M + R combination. The average zones of inhibition of quercetin against MRSA clinical isolates were 13.33 ± 0.26 mm while it was 13.5 ± 0.21 mm in case of ATCC 43300. The results revealed that quercetin alone possessed antibacterial activity while morin and rutin have the same activity when they were used in combination. It was also found to increase the activity of antibiotics AMP, CEPH, CET and ME that experienced resistance. Quercetin also had a more blunting effect for CIP and LEV in comparison to M + R. It is evident from data presented in Table 4 that among the antibiotics facing resistance, CET was most responsive in combination with quercetin as zones of inhibition enhanced from 0 to 20.5 ± 0.5 mm against ATCC 43300 and 20.83 ± 0.45 mm in case of clinical isolates, respectively. IMP was active against test MRSA and its activity was further improved by quercetin from 16 ± 0.90 mm to 22 ± 1.10 mm against ATCC 43300 while in case of clinical isolates it increased from 16.18 ± 0.92 mm to 22.18 ± 1.12 mm. ERY and VAN activities remained unchanged and resistance for S-T persisted even when it was combined with quercetin.

As quercetin and M + R were found to have activities against MRSA, they were combined together, and tested in flavonoids combination as well as flavonoids-antibiotic combinations against test bacteria. The results in the Table 4 show that combined effect of flavonoids is greater than their individual effects, zones of inhibition of these flavonoids (M + R + Q) against ATCC 43300 were 16.5 ± 0.21 mm and for clinical isolates 16.23 ± 0.26 mm, which are greater than that of M + R (11.5 ± 0.22 mm for ATCC 43300 & 11.58 ± 0.21 mm against clinical isolates) and quercetin (13.33 ± 0.21 mm & 13.5 ± 0.26 mm) respectively. It is evident from the results in Table 4 that with triplet combination (M + R + Q) the activity of both AMP and AMO were increased. The inhibitory zones observed in case of AMP and AMO with M + R + Q were 22.5 ± 1.2 mm & 23.50 ± 1.1 mm against ATCC 43300. While against clinical isolates zone of inhibition of AMO in combination with M + R + Q was 23.73 ± 1.1 mm which was greater than that observed for AMO against the clinical isolates in combination with M + R (14.18 ± 0.36 mm) and Q (13.33 ± 0.26 mm). Same trend was also observed in case of AMP in combination with M + R + Q, where inhibitory zones against the clinical isolates were 22.63 ± 1.2 mm which were greater than that observed for AMP when combined with M + R and Q. M + R + Q showed no effect on the activity of VAN and ERY as the zones of inhibition remained same. The antagonistic effect of M + R + Q on CIP and LEV was greater than that observed with M + R and Q alone. M + R + Q had no effect on the activities of S-T and CEF.

### MICs by serial half dilution method

Test flavonoids in combination or alone were quantified for activities using serial broth half dilution method (Table 5). The results for clinical isolates showed variation with 14 isolates giving MIC of 400 + 400 μg/ml for M + R while rest of isolates (n = 86) were inhibited at 800 + 800 μg/ml concentrations. Similarly, for M + R + Q, 60 isolates gave MIC of 200 + 300 + 300 μg/ml while remaining isolates (n = 40) were inhibited at 200 + 600 + 600 μg/ml concentrations. The MIC of quercetin determined by serial half dilution method against the MRSA 43300 was 300 μg/ml and same MIC was observed against 64 MRSA clinical isolates shown in Table 5. While against remaining clinical isolates n = 36, the MIC of quercetin was 600 μg/ml.

**Table 5:** **MICs of flavonoid/(s) against** ***S. aureus*** **(ATCC 43300) and clinical isolates of MRSA**

**Flavonoids**	**†MIC (μg/ml)**	*MIC (μg/ml)
M + R	400 + 400	400 + 400 (n = 14)
		800 + 800 (n = 86)
Q	300	300 (n = 64)
		600 ( n = 36)
M + R+ Q	200 + 300 + 300	200 + 300 + 300 (n = 60)
		200 + 600 + 600 (n = 40)

† activity against *S. aureus* (ATCC 43300).

*activity against clinical isolates.

### Exact MICs of flavonoids and flavonoids-antibiotics by incremental increase approach

Exact MICs of M + R, quercetin and M + R + Q alone and in combination with antibiotics were determined against MRSA clinical isolates and ATCC 43300. The MIC of M + R first determined by half dilution method is given in Table 5. It is evident that MIC of each flavonoid in combination was 400 μg/ml against standard strain ATCC 43330. However, in case of clinical isolates MIC of 14 strains was 400 μg/ml while rest of 86 isolates gave MIC value of 800 μg/ml. In order to arrive at exact MIC for these strains an incremental increase approach was adopted. The MIC data from this method is presented in Table 6 for flavonoid and their combinations while Table 7 gives exact MICs of flavonoids in combination with test antibiotics.

**Table 6:** **Exact MICs (μg/ml) of flavonoid/(s) against** ***S. aureus*** **(ATCC 43300) and clinical isolates of MRSA**

	**(M + R)** ^**♣**^	**Q**	**(M + R)** ^**♣**^ **+ Q**
*S. aureus* (ATCC 43300)	400	260	(280)^♣^ + 140
Clinical Isolates	427.40^a^ ± 14.40	279.00^a^ ± 14.65	(303.56)^**♣**^ ±16.74 + 163.56^a^ ±15.23
MIC Ranges	400 - 440	260 – 300	M + R = 280 – 340
			Q = 140 – 200

^♣^= MIC’s of M & R are same in combination.

^a^= Average value.

**Table 7:** **Exact MICs (μg/ml) of flavonoid/(s) with antibiotics against** ***S. aureus*** **(ATCC 43300) and clinical isolates of MRSA**

	**With (M + R)** ^**♣**^	**With Q**	**With (M + R)** ^**♣**^ **+ Q**
**AMO†**	**340**	**260**	**(140)** ^**♣**^ **+ 80**
AMO*	367.40 ± 15.71	279.00^a^ ± 14.65	(163.56^a^)^**♣**^ ±16.75 + 103.56^a^ ± (15.99)
MIC range*	340 - 380	260 - 320	M + R = 140 – 200; Q = 80 – 140
**AMP†**	**400**	**160**	**(160)** ^**♣**^ **+ 80**
AMP*	427.40^a^ ± 14.40	178.80^a^ ± 14.25	(186.56^a^)^**♣**^ ± 15.80 + 112.37^a^ ± 16.42
MIC range*	400 - 440	160 - 200	M + R = 160–220; Q = 80 – 140
**CEPH†**	**340**	**160**	**(140)** ^**♣**^ **+ 60**
CEPH*	367.40 ± 15.71	178.80^a^ ± 14.25	(163.56^a^)^**♣**^ ± 16.52 + 83.56^a^ ±15.02
MIC range*	340 - 380	160 - 200	M + R = 140 – 200; Q = 60 – 120
**CET †**	**320**	**140**	**(140)** ^**♣**^ **+40**
CET*	347.40 ± 12.92	158.60^a^ ± 15.11	(162.94^a^)^**♣**^ ± 16.98+ 65.94 ^a^ ± 16.9
MIC range*	320 - 360	140 - 180	M + R = 140 – 180; Q = 40 -100
**IMP†**	**300**	**120**	**(120)** ^**♣**^ **+40**
IMP*	327.80^a^ ± 14.85	138.80^a^ ± 14.99	(148.31^a^)^**♣**^ ± 19.59+ 66.56^a^ ±16.50
MIC range*	300 - 340	120 - 160	M + R = 120–180 ; Q = 40 – 100
**ME†**	**360**	**180**	**180 + 80**
ME*	387.40 ± 15.78	196.70^a^ ± 12.75	(208.51^a^)^**♣**^ ± 19.98 + 88.51^a^ ±19.80
MIC range*	360 - 400	180 - 220	M + R = 180 – 240; Q = 80-160

^♣^MIC of M & R is same.

^a^= Average value.

*against MRSA clinical isolates (n = 100).

† against *S. aureus* (ATCC 43300).

Exact MIC for M + R was 400 μg/ml against ATCC 43300 while for clinical isolates (n = 100) average MIC was 427.40 ± 14.40 μg/ml (Table 6). When combined with amoxicillin (AMO) the MIC of M + R decreased to 340 μg/ml against ATCC 43300. This indicated that there may be synergism or additive relationship between the flavonoids and antibiotics experiencing resistance. Similarly average MIC of M + R (427.40 ± 14.40 μg/ml) was reduced to 367.40 ± 15.71 μg/ml when combined with AMO. In case of CET the MIC of M + R against ATCC 43300 was found to reduce from 400 μg/ml to 320 μg/ml while against the clinical isolates it decreased from 427.40 ± 14.40 μg/ml to 347.40 ± 12.92 μg/ml. Similar trend was also observed in case of other antibiotics. A difference in the MIC’s of M + R and M + R+ antibiotic can be seen in Tables 6 & 7. It is evident that reduction in MICs of M + R is greater with IMP as it dropped from 400 μg/ml to 300 μg/ml followed by CET, AMO and CEPH with MICs reducing to 320 μg/ml, 340 μg/ml and 340 μg/ml respectively against ATCC 43300. Same trend was also observed with these antibiotics when tested against clinical isolates. Methicillin was found to be less responsive than other antibiotics. The reduction in MIC of flavonoids when combined with antibiotics was an indication of their increased activity against the bacteria under study.

The MIC of quercetin determined by serial half dilution method against ATCC 43300 was 300 μg/ml and similar MIC was observed against 64 MRSA clinical isolates (Table 6), while remaining clinical isolates (n = 36) were inhibited at 600 μg/ml. Exact MIC of quercetin came to be 260 μg/ml for ATCC 43300 and for clinical isolates (n = 100) it varied between 260 – 300 μg/ml. Reduction in MIC of quercetin in combination with antibiotics was observed (Table 7) as it dropped from 260 μg/ml to 160 μg/ml when combined with AMP in case of MRSA 43300 whilst average MIC against clinical isolates reduced from 284.64 ± 19.31 μg/ml to 184.64 ± 16.25 μg/ml. Average MIC of Q with CEPH against clinical isolates reduced to the same extent as for AMP. For ME the decrease in the MIC was less than the other antibiotics.

Combinations of M + R + Q were tested to determine the effectiveness against test bacteria. A clear reduction in MICs was evident in comparison to their individual MICs. The MIC of M + R + Q came to 300 + 300 + 200 μg/ml for sixty clinical isolates while it was 600 + 600 + 200 μg/ml for rest of forty isolates. Exact MIC of M + R + Q was also determined (Table 6). It was observed that exact MIC of Q in combination was 140 μg/ml and that of M + R was 280 + 280 μg/ml against ATCC 43300 whilst in case of clinical isolates (n = 100) Q had average MIC 163.56 ± 15.23 μg/ml while M + R had (303.56 ± 16.74 μg/ml) + (303.56 ± 16.74 μg/ml). It is evident from data that in case of M + R + Q MICs of not only rutin-morin reduced but quercetin’s MIC dropped as well. The MIC of rutin-morin each was 400 μg/ml but along with qurecetin it fell to 280 μg/ml against ATCC 43300 whilst that of qurecetin reduced from 260 μg/ml to 140 μg/ml. Thus the antibacterial activity of flavonoids was found to increase against MRSA when used in combination with each other. Similarly the incorporation of antibiotics with M + R + Q had an enhancing effect on activities of test antibiotics. Data in Table 7 suggests that MIC of this combination was further reduced when combined with test antibiotics, i.e., with AMO, the MIC of rutin-morin and quercetin was further reduced to 140 μg/ml (each) and 80 μg/ml against ATCC 43300. The average MIC of rutin-morin with AMO against the MRSA clinical isolates reduced to 163.56 ± 16.75 μg/ml from 367.40 ± 15.71 μg/ml but quercetin had no effect on AMO activity. However in M + R + Q with AMO, quercetin activity was increased as its average MIC decreased to 103.56 ± 15.99 μg/ml from 284.64 ± 19.31 μg/ml against clinical isolates. The reduction in average MIC of M + R in M + R + Q combination from 367.40 ± 15.71 to 148.31 ± 19.59 μg/ml with IMP found greater than the rest of antibiotics against clinical isolates. While a decrease in average MIC of quercetin in M + R + Q combination, from 164.44 ± 17.11 μg/ml to 65.94 ± 16.9 μg/ml with CET was more than any other test antibiotics against clinical isolates. However, ME showed least activity with this flavonoid combination. Important mention in this flavonoids combination is its enhancing effect on both AMP and AMO, while the rest of the flavonoids are either increasing AMO or AMP activity but not both.

MIC’s of antibiotics alone as well as in combination with flavonoids (Table 8) were determined in order to prove that flavonoids decreased the MICs of antibiotics thereby increasing their activity against MRSA. In combination with flavonoids all antibiotics encountering resistance became sensitive while maximum susceptibility was conferred upon cell wall synthesis inhibitors. From Table 8 it is evident that MIC of AMO was 256 μg/ml, which reduced significantly to 64 μg/ml in combination with M + R against ATCC 43300. The MIC of AMO in combination with M + R + Q decrease more significantly than M + R from 256 μg/ml to 8 μg/ml. The observed average MIC of AMO against clinical isolates (n = 100) was 197.70 ± 64.02 μg/ml that became 49.43 ± 16.03 μg/ml when it was combined with M + R; and it reduced further to 6.00 ± 2.13 μg/ml when used with M + R + Q. MIC of AMP was 128 μg/ml against ATCC 43300 and its average MIC value against clinical isolates was 162.85 ± 68.05 μg/ml. However, no effect was observed in case of AMP when it was combined with M + R. In combination with Q, MIC of AMP dropped significantly from 128 μg/ml to 16 μg/ml. The highest increase in activity was observed when AMP was used in combination with M + R + Q, with MIC dropping from 128 μg/ml to 4 μg/ml against ATCC 43300 while its average MIC against clinical isolates (n = 100) reduced from 162.85 ± 68.05 μg/ml to 5.09 ± 2.13 μg/ml.

**Table 8:** **Exact MICs (μg/ml) of antibiotics with flavonoid/(s) against** ***S. aureus*** **(ATCC 43300) and clinical isolates of MRSA**

	**Antibiotic alone**	**With M + R**	**With Q**	**With M + R + Q**
**AMO †**	**256**	**64** ^**♣**^	**256**	**8**
AMO *	197.70^a^ ± 64.02	49.43^a^ ± 16.03	197.70^a^ ± 64.02	6.00^a^ ± 2.13
MIC range*	128 – 256	32 - 64	128 - 256	2-8
**AMP †**	**128**	**128**	**16**	**4**
AMP *	162.85^a^ ± 68.05	162.85^a^ ± 68.05	20.36^a^ ± 8.51	5.09^a^ ± 2.13
MIC range*	64 – 256	64 - 256	8-32	2-8
**CEPH†**	**256**	**64**	**32**	**8**
CEPH*	200.96 ^a^ ± 63.69	50.38 ^a^ ± 17.92	25.12^a^ ± 7.92	6.28^a^ ± 1.99
MIC range*	128 – 256	32 - 64	16 - 32	4-8
**CET †**	**64**	**16**	**8**	**2**
CET*	67.52 ^a^ ± 30.48	27.09 ^a^ ± 16.94	8.44^a^ ± 3.81	2.11^a^ ± 0.95
MIC range*	32 – 128	16 - 64	4 - 16	1-4
**IMP †**	**32**	**8**	**4**	**1**
IMP*	130.88 ^a^ ± 84.02	32.72 ^a^ ± 16.15	19.88^a^ ± 10.51	4.97^a^ ± 2.63
MIC range*	32 – 256	8 - 64	4 - 32	1-8
**ME †**	**64**	**16**	**8**	**2**
ME*	135.68 ^a^ ± 54.03	33.92 ^a^ ± 13.51	16.96^a^ ± 6.75	4.24^a^ ± 1.69
MIC range*	64 – 256	16 - 64	8 - 32	2-8

^♣^MIC of M & R is same.

^a^= Average value.

*against MRSA clinical isolates (n = 100).

† against *S. aureus* (ATCC 43300).

### Fractional inhibitory concentration (FIC) & Fractional inhibitory concentration index (FICI)

In order to term effect of antibiotics used in combination with flavonoids as synergistic or additive FICI’s were evaluated. The results (Table 9) showed an additive response in case of quercetin and morin + rutin in combination with test antibiotics such as amoxicillin, Ampicillin, ceftriaxone, cephradine, imipenem and methicillin. However, synergism was indicated when these antibiotics were used in conjunction with M + R + Q.

**Table 9:** **Fractional Inhibitory Concentration indices (FICI) of flavonoid/(s) and antibiotics against** ***S. aureus*** **(ATCC 43300) and clinical isolates of MRSA**

**Flavonoid/(s) + antibiotics**	**FICI**		
	***S. aureus*** **(ATCC 43300)**	**MRSA clinical isolates (n = 100)**	**Inference**
M + R + AMO	0.9	0.9	Additive
M + R + CEPH	0.9	0.95	Additive
M + R + CET	0.8	0.94	Additive
M + R + IMP	0.84	0.85	Additive
M + R + ME	0.95	0.97	Additive
Q + AMP	0.74	0.77	Additive
Q + CEPH	0.74	0.77	Additive
Q + CET	0.66	0.69	Additive
Q + IMP	0.66	0.69	Additive
Q + ME	0.82	0.83	Additive
M + R + Q + AMO	0.59	0.66	Additive
M + R + Q + AMP	0.59	0.68	Additive
M + R + Q + CEPH	0.46	0.50	Synergism
M + R + Q + CET	0.31	0.44	Synergism
M + R + Q + IMP	0.32	0.45	Synergism
M + R + Q + ME	0.45	0.5	Synergism

### Detection of cytoplasm membrane damage

The potassium leakage was measured for test bacteria with flavonoids & antibiotics alone and flavonoids-antibiotics in combination. From the data (Tables 10 and 11) it is apparent that K+ leakage from flavonoid-antibiotic combination was greater than the flavonoids and antibiotics alone. All antibiotics and flavonoids induced release of K+ confirming damage they inflicted to bacterial cell membrane. K+ measured in case of AMO was 25.7 ppm for ATCC 43300 whilst for clinical isolates average K+ release was 25.79 ± 0.16 ppm. AMO’s K+ release in combination with M + R was 32.3 ppm and 32.40 ± 0.13 ppm for ATCC 43300 and clinical isolates, respectively. Highest leakage of potassium was observed for IMP that was 26.6 ppm against ATCC 43300 and 26.79 ± 0.14 ppm for clinical isolates. The K+ leakage was further increased when IMP was used with flavonoids i.e. 36.6 ppm with M + R, 39.2 ppm with Q and 44.7 ppm with M + R + Q against ATCC 43300. The effect became even more when M + R + Q was combined with antibiotics (amoxicillin, Ampicillin, ceftriaxone, cephradine, imipenem and methicillin), since K+ level from M + R + Q was 32.7 ppm in case of ATCC 43300 while in case of isolates average value of K+ leakage was 32.29 ± 0.13 ppm. K+ leakage for AMO in combination with M + R was 32.3 ppm, which increased to 41 ppm with M + R + Q in case of ATCC 43300. Similar trend was observed in case of other antibiotics.

**Table 10:** **Potassium leakage (ppm) by flavonoid/(s) against** ***S. aureus*** **(ATCC 43300) and clinical isolates of MRSA**

	**Control**	**Q**	**M + R**	**(M + R)** ^**♣**^ **+ Q**
***S. aureus*** **(ATCC 43300)**	10.2	28.4	26.4	32.7
Clinical Isolates	10.19 ± 0.18	28.49 ± 0.14	26.49 ± 0.12	32.29 ± 0.13

^♣^MIC of M & R is same.

**Table 11:** **Potassium leakage (ppm) by flavonoid/(s) with antibiotics against** ***S. aureus*** **(ATCC 43300) and clinical isolates of MRSA**

**Test antibiotics**	**Antibiotic alone**	**Antibiotic + M + R**	**Antibiotic + Q**	**Antibiotic + M + R + Q**
**AMO †**	25.7	32.3	NT	41
AMO *	25.79 ± 0.16	32.40 ± 0.13	NT	41.09 ± 0.11
**AMP †**	25.6	NT	34.4	39.6
AMP *	25.69 ± 0.13	NT	34.49 ± 0.14	39.83 ± 0.12
**CEPH†**	25.70	33.20	35.2	40
CEPH*	25.89 ± 0.14	33.30 ± 0.14	35.39 ± 0.14	40.10 ± 0.10
**CET †**	25.9	34.60	37.5	42.6
CET*	25.96 ± 0.10	34.69 ± 0.15	37.59 ± 0.10	42.69 ± 0.13
**IMP †**	26.6	36.6	39.2	44.7
IMP*	26.79 ± 0.14	36.79 ± 0.15	39.26 ± 0.14	45.89 ± 0.14
**ME †**	25.1	31.4	34.3	39.2
ME*	25.29 ± 0.12	31.52 ± 0.13	34.43 ± 0.11	39.33 ± 0.12

*against MRSA clinical isolates (n = 100).

† against *S. aureus* (ATCC 43300).

NT = Not tested.

## Discussion

MRSA is now commonly isolated bug from nosocomial infections and has potential to lead to fatalities. With passage of time MRSA has also shown resistance to other antibiotics as well such as tetracyclines, erythromycin and genatmacin [17]. As a result of MDR (multidrug resistance) the only choice left is vancomycin, which is also experiencing resistance and reports of emergence of vancomycin intermediate *S.aureus* (VISA) and vancomycin resistant *S. aureus* (VRSA) are there [17]. Therefore it is the need of day to analyze MRSA and find new treatment modalities.

Morin and rutin alone have no antibacterial activity but together they were active against *S. aureus* ATCC 25923 and E. coli ATCC 25922 [18]. Moreover, rutin has been reported to enhance antibacterial activity of several compounds such as aminopenicillanic acid [19] and other flavonoids such as morin and rutin against *Salmonella enteritidis* and *Bacillus cereus* [15].Morin was found active *E. coli* ATCC 25922, *P. aeruginosa* ATCC 27853 and *S. aureus* ATCC 29213 and respective clinical isolates [20].

Quercetin activity has also been reported to increase with oxacillin, vancomycin, gentamycin, and erythromycin [21]. Quercetin is also found to increase the activity of rifampicin and fusidic acid against MRSA 43300 and clinical isolates [22]. Quercetin alone has been found active against *S. aureus* and *K. pneumoniae* [23]. It has also been found to be potentiating effects of antibiotics such as rifampicin, fusidic acid and rifampicin against MRSA and MSSA [24]. Quercetin alone and in combination with gentamycin, levolfloxacin and sulphadiazine was found to be synergistic since MIC of qurecetin and test antibiotics decreased four folds when they were combined with each other [14]. Quercetin’s MIC of 260 μg/ml is comparable to previous report of 256 μg/ml against MRSA [7]. It is evident from data (Table 4) that among resistant antibiotics on which quercetin had enhancing effect; CET was most responsive as increase in the activity was greater than other antibiotics i.e. zones of inhibition enhanced from 0 to 20.5 ± 0.5 mm in ATCC 43300. While in case of clinical isolates (n = 100) the average zone of inhibition increased from 0 to 20.83 ± 0.45 mm. Those antibiotics including ERY, VAN and IMP which were sensitive against ATCC 43300, among them only IMP antibacterial activity increased as its inhibitory zone rose from 16 ± 0.90 mm to 22 ± 1.10 mm in combination with quercetin. The S-T remained resistant even in combination with quercetin. The results are consistent with different reports in which it was used in combination with antibiotics. Results can also be compared to the results reported by Goyal et al., [25] where morin was used in combination with quercetin against *S. aureus*, *Salmonella typhi, E. coli* and *B. subtilis*. It was found that as a result of using them in combination MICs of these flavonoids had significantly decreased e.g. MIC’s of morin and quercetin against *S. aureus* were 64 μg/ml and 32 μg/ml. But when these flavonoids were combined MIC became 16 μg/ml [15]. Morin and rutin alone had no activity against MRSA but in combination showed antibacterial activity against said bacteria. In similar studies flavonoids combinations and their metallo-combinations show activity against various microbes [26,27]. Morin alone has been found to be active against *Salmonella enteritidis* with MIC of 150 μg/ml and *B. cereus* with MIC of 300 μg/ml. MICs were further reduced when morin was combined with rutin, i.e. MIC of morin with rutin lessened to 50 μg/ml against *S. enteritidis*, while in case of *B. cereus* morin’s MIC reduced to 100 μg/ml [15]. Morin also showed inhibitory activity against *S. typhi, E. coli, B. subtilis*, and *S. aureus* with MIC’s of 128 μg/ml, 128 μg/ml, 64 μg/ml and 64 μg/ml, respectively [25]. Morin when tested against *P. aeruginosa* (ATCC 9027), *S. epidermidis* (ATCC 12228), B. subtilis (ATCC 9372 & 6633) and *E. coli* (ATCC 8739 & 11775) inhibited the growth of all these bacteria [28]. In another study the activity of morin and its complexes with Gadolinium and Lutetium were tested against different bacterial strains such as *E. coli, K. pneumoniae , S. aureus*; results revealed morin complexes with Lu, and Gd, to be more active than alone, thus suggesting potentiation by these elements [29]. As evident from literature, morin antibacterial activities enhance in combination with rutin, therefore, this combination was tested against MRSA and also with conventional antibiotics that are experiencing resistance from this bug.

In present study combination of three flavonoids enhanced their anti staphylococcal potential which is evident from the MIC data. The MIC of M + R decreased from 400 + 400 μg/ml to 280 + 280 μg/ml and that of Q reduced from 260 μg/ml to 140 μg/ml against MRSA 43300. In case of clinical isolates the M + R average MICs reduced from 427.40 ± 14.40 μg/ml to 303.56 ± 16.74 μg/ml. While the average MIC of Q against the clinical isolates decreased from 279.00 ± 14.65 μg/ml to 163.56 ± 15.23 μg/ml. It is in confirmation to the studies in which rutin, morin and quercetin gave a synergistic response against *S. aureu, E.coli* and *Enterobacter aerogenes* [7,19,30].

In present study MIC of CEPH was 256 μg/ml against ATCC 43300 and its average MIC was 200.96 ± 63.69 μg/ml against clinical isolates. The MIC of CEPH in combination with M + R decreased significantly to 64 μg/ml against the ATCC 43300 and its average MIC against clinical isolates decreased to 50.38 ± 17.92 μg/ml. The MIC of CET was 64 μg/ml against ATCC 43300 that reduced to 16 μg/ml when it was combined with M + R. While in case of clinical isolates average MIC of 67.52 ± 30.48 μg/ml for CET was observed that reduced to 27.09 ± 16.94 μg/ml when it was combined with M + R. Similarly, CET in combination with Q and Q + M + R, against ATCC 43300 MIC of 64 μg/ml dropped to 32 μg/ml and 8 μg/ml respectively that are in confirmation to earlier reports [31,32].

The MIC of imipenem was 32 μg/ml against ATCC 43300 that reduced to 8 μg/ml when IMP was combined with M + R. While its average MIC against clinical isolates was 130.88 ± 84.02 μg/ml. In combination with M + R average MIC reduced to 32.82 ± 16.15 μg/ml. The MIC range (32 - 256 μg/ml) of IMP against MRSA clinical isolates found almost similar to that reported earlier [33]. In this study IMP tested alone and in combination with catechins flavonoids (extracted from green tea leaves) against MRSA clinical samples and standard ATCC 25923. The MIC range found in this study was 16 – 256 μg/ml. In present study reduction in the imipenem’s MIC along with Q + M + R was found greater than the other flavonoids. The MIC observed against ATCC 43300 was 1 μg/ml and against the clinical isolates it’s MIC range reduced from 32 - 256 μg/ml to 1–8 μg/ml.

MIC of ME was 64 μg/ml against ATCC 43300 that reduced to 16 μg/ml when it was combined with M + R, this trend was also observed in case of clinical isolates where average MIC decreased form 135.68 ± 54.03 μg/ml to 33.92 ± 13.51 μg/ml. Effective concentrations of ME were further reduced against the test bacteria when it was combined with M + R + Q with MICs of 2 μg/ml and 4.24 ± 1.69 μg/ml for ATCC 43300 and clinical isolates respectively.

The results also revealed that combined effects of morin + rutin and quercetin in combination with the antibiotics were additive (FICI <1). However, relationships between quercetin + morin + rutin and CEPH, IMP, CET and ME were synergistic (FICI ≤ 0.5). While Q + M + R with AMP and AMO showed additive effect (FICI <1). These results are in in conformity with earlier findings where quercetin was found to be synergistic with minocycline, fusidic acid and rifampicin [24].

Various compounds e.g. phenolics, have been known for their ability to effect cytoplasmic membrane permeability consequently resulting in leakage of cellular constituents like nucleic acids, proteins and inorganic ions such as phosphate and potassium [34]. Results of this study suggest potassium leakage when flavonoids were used alone, in combinations and with test antibiotics. Potassium leakage data for Q (28.4 ppm), M + R (26.4 ppm), and M + R + Q (32.7 ppm) against ATCC 43300 suggest increase in extracellular K+ in comparison to control (10.2 ppm). The highest K+ release was observed in case of antibiotics in combination with M + R + Q. The results can be paralleled to that of galangin, a flavonol that target cytoplasmic membrane of *S. aureus* and cause potassium leakage [6].

Since the test concentrations used for antibiotics were their MICs, therefore, K+ release was also observed in the inoculums of test bacteria to which antibiotics were added. The K+ release was increased when flavonoids were used in conjunction to test antibiotics and highest release was found in case of CET + M + R (34.60 ppm & 34.69 ± 0.15 ppm), CET + Q (37.5 ppm & 37.59 ± 0.10 ppm), CET + M + R + Q (42.6 ppm & 42.69 ± 0.13 ppm) against ATCC 43300 and clinical isolates, respectively. Similarly, IMP + M + R (36.6 ppm & 36.79 ± 0.15 ppm), IMP + Q (39.2 ppm & 39.26 ± 0.14 ppm), IMP + M + R + Q (44.7 ppm & 42.89 ± 0.14 ppm) against ATCC 43300 and clinical isolates, respectively. Since both CET and IMP are bacterial cell wall inhibitors, therefore, it can be hypothesized that wall damage was implicated by test antibiotics while cytoplasmic membrane injury was inflicted by flavonoids; with greater damage observed with combination of flavonoids.

The present data suggests that antibiotics activity was increased in combination with flavonoids. The flavonoids used such as morin, rutin, and quercetin may also target the cell wall of MRSA as evidenced from potassium leakage data. Thus, combination of flavonoids with antibiotics can be considered for therapeutic application in case of microbes expressing resistance after safety evaluations.

The present research was limited to in-vitro studies only because of non-availability of animal models facilities, which remained the major limitation of these studies. Since the findings are promising, therefore, they can be extended to in-vivo stage.

## Conclusions

From the antibiotic sensitivity assays it is evident that morin and rutin in combination were effective against MRSA ATCC 43300 and its clinical isolates, while quercetin alone found active against test bacteria. The activity of flavonoids was further enhanced against ATCC 43300 and clinical isolates, when rutin, morin and quercetin were used in combination. Test bacteria responded to ampicillin, amoxicillin, cephradine, methicillin and ceftriaxone when these antibiotics were mixed with flavonoids. Similarly, imipenem activity was further increased against test MRSA strains when combined with flavonoids. Through MIC assays it was observed that both the flavonoids and antibiotics supplemented each other effects. FICI results indicated an additive relationship between the flavonoids and the antibiotics. While a synergistic effect was also observed in M + R + Q-antibiotics combinations. The cell wall synthesis inhibitors showed positive activity in conjunction to test flavonoids while activity of antibiotics affecting nucleic acid got blunted. Measurement of potassium loss suggested cytoplasmic membrane damage in conjunction with cell wall damage can be assumed to be the mechanism of action of these flavonoids antibiotic combinations. The sum of the substance of results was that the activity of both flavonoids and antibiotics found to increase when they were combined with each other.
